# Polio Data Quality Improvement in the African Region

**DOI:** 10.29245/2578-3009/2021/S2.1105

**Published:** 2021-04-16

**Authors:** Bernard Ntsama, Ado Bwaka, Reggis Katsande, Regis Maurin Obiang, Daniel Rasheed Oyaole, Pascal Mkanda, Joseph Okeibunor

**Affiliations:** 1 Data Manager, IST West Africa; 2 IVD/PEP Focal Point, IST West Africa; 3 Data Manager AFRO; 4 Data Manager, IST Central Africa; 5 Data Manager, WHO Nigeria

**Keywords:** Polio eradication, Data quality improvement, Data availability, Monitoring, Decision making, Surveillance performance indicators, Innovative technologies

## Abstract

The polio Eradication Initiative (PEI) is one of the most important public health interventions in Africa. Quality data is necessary to monitor activities and key performance indicators and access year by year progress made. This process has been possible with a solid polio health information system that has been consolidated over the years.

This study describes the whole process to have data for decision making. The main components are the data flow, the role of the different levels, data capture and tools, standards and codes, the data cleaning process, the integration of data from various sources, the introduction of innovative technologies, feedback and information products and capacity building. The results show the improvement in the timeliness of reporting data to the next level, the availability of quality data for analysis to monitor key surveillance performance indicators, the output of the data cleaning exercise pointing out data quality gaps, the integration of data from various sources to produce meaningful outputs and feedback for information dissemination.

From the review of the process, it is observed an improvement in the quality of polio data resulting from a well-defined information system with standardized tools and Standard Operating Procedures (SOPs) and the introduction of innovative technologies. However, there is room for improvement; for example, multiple data entries from the field to the surveillance unit and the laboratory. Innovative technologies are implemented for the time being in areas hard to reach due to the high cost of the investment.

A strong information system has been put in place from the community level to the global level with a link between surveillance, laboratory and immunization coverage data. To maintain standards in Polio Information system, there is need for continuous training of the staff on areas of surveillance, information systems, data analysis and information sharing. The use of innovative technologies on web-based system and mobile devices with validation rules and information check will avoid multiple entries.

## Introduction

In 1988, the World Health Assembly (WHA) declared polio a critical public health problem^1^. An important aspect of polio eradication addressed in WHO policy resolutions is the need to improve programme monitoring and evaluation at the national, regional and global levels.

The Polio Eradication and Endgame Strategic Plan 2013-2018 highlighted programme monitoring as a crucial element towards attaining its objectives^2^. Equally highlighted in these resolutions and programme policy documents is the need to improve national disease surveillance systems for rapid detection, response and control of outbreaks including thorough investigation and confirmation of clinical diagnoses of poliomyelitis through serological and virus isolation techniques.

To monitor disease trends in polio eradication, an electronic data information system was put in place with the aim of collecting data from surveillance activities carried out in the field to detect wild polio viruses or vaccine derived polioviruses^3^.A structured network of data flow has been established from community, health facilities, district, and national level to WHO^4^. This structure gives responsibility and visibility to each level of the network for accountability and troubleshooting. The information generated are used at all levels by programme managers, partners, donors and the public^5^.

Tremendous achievements have been recorded by the Polio Eradication programme particularly in the data management components, where emphasis is placed on timely availability of data, data quality and completeness of reporting. The quality of surveillance data has contributed directly to the decrease in the number of endemic countries from 125 countries in 1988 to 2 countries (Afghanistan and Pakistan) in 2018. The quality of polio data in the African region has improved over time and has led to appropriate planning of activities and timely interventions. However, these achievements have not been systematically documented.

This paper documents and describes efforts, mechanisms and tools put in place to generate data for decision making as well as the results produced. It highlights potential issues that need to be addressed in the critical phase of the Polio eradication endgame in Africa and highlights the potential of the introduction of new technologies in improving planning and monitoring of public health interventions that share similarities with polio eradication.

## Methods

### Design

A descriptive study design was used and was designed to facilitate a description and analysis of special and innovative practices that were employed to achieve the desired targets in data management for polio eradication in the African Region. Data collection was based on review of documents, including programme reports and Standard Operating Procedures as well as observations and experiences by system users. Data collection was aided by an in-depth triangulation of data management practices that contributed to the interruption of the transmission of polio viruses in the African Region.

Quantitative approach was used to track wild poliovirus trends in African Region through the existing information system for proper interventions.

Basic analysis were performed on AFP database in order to determine high risks areas, to monitor the surveillance performance indicators and to detect information gaps.

## Description of data improvement strategies

### The data flow and the role of the different system levels

The polio data information system is initiated with the detection of cases of Acute Flaccid Paralysis (AFP) where a form is completed, and 2 stool specimens collected 24 hours apart and sent to the laboratory. The laboratory and surveillance services have the responsibility of recording the case information while waiting for lab results. Each week, the national level shares AFP database (both surveillance and laboratory) with their respective WHO country office (WCO) which, in turn, shares the same data with the WHO Inter-Country Support Team Office (IST). WCO and IST verify the completeness of data before sharing with WHO Regional Office for Africa (WHO/ AFRO). Intercountry Support Team (IST) monitors also the timeliness of reporting AFP data from countries to IST.

The Global Polio Eradication Initiative (GPEI) has built a strong polio laboratory network which comprises national level laboratories, inter-country laboratories, regional reference laboratories and global laboratories. The Global Polio Laboratory Network (GPLN) was established in 1990 by WHO and national governments. Its primary responsibility is to distinguish poliovirus as a cause of acute flaccid paralysis (AFP) from AFP caused by other diseases.

National laboratories receive AFP samples from the field and either process them if the country has a WHO accredited polio laboratory or send them to the inter-country polio laboratories or regional reference laboratories. In West Africa sub-region, there are six national/inter-country laboratories and one regional reference laboratory. Staff are trained regularly in each of the laboratories and the laboratories undergo an accreditation exercise using WHO guidelines and tools.

### Data capture and tools

For all countries in the African Region, standardized data collection tools have been developed. A standard Epi-Info database has been designed and implemented at national level. For each AFP case detected, information is recorded in the national database. Geo-coordinates are taken and reflected in the investigation form for each AFP case investigated. For large countries such as Nigeria, there is a decentralized data entry at zonal level and, prior to data sharing with the IST, data is merged at national level. At the same time, a polio laboratory database has been developed and for all AFP cases, information is captured on specimens collected in the polio laboratory database.

Data sources are AFP database from Surveillance units of the Ministry of health and polio laboratories, routine EPI coverage data, ISS data generated from server of the GIS Centre at the WHO AFRO Regional Office. ISS visits are carried out individually or jointly by MOH and partners.

### Standards and codes

For standardization of the data management system, it has been agreed to have the patient identification number (EpidNumber) in a standardized format. This format is a combination of Country Code, Region code, District code, the year and the case number. For calculation of indicators and the generation of the patient Number, demographic data is collected at the beginning of the year and saved in a utility database. The utility database is composed of Country, region/province, their codes and their target population.

### Data cleaning process

Data cleaning begins at country level after the collection of data. The national data manager is responsible for assessing data quality and completeness and identifying errors. The data cleaning programs within the AFP data processing module help the data managers. Most data errors, inconsistencies and incompleteness are rectified during the monthly/quarterly data harmonization meeting where all the actors that are part of the data system meet to discuss and correct data issues. At IST level, feedback is provided to countries on errors and discrepancies observed in data received. The same data quality assessment is also carried out by AFRO.

### Integration of data from different sources

Data from 3 data sources are used in a complementary manner to monitor programme progress: AFP surveillance data, the lab data and routine immunization data. While the patient’s number is used to link surveillance and lab data, the district name and/or district code is used to link immunization data with surveillance data. With the patient’s number, it is possible to correlate the lab results and the district name or district code showing the location of the case and the administrative vaccination coverage for the district.

### Introduction of innovative technologies in the data management domain

The number of wild polio viruses decreased in Africa Region to reach 0 case in 2015. Unexpectedly, in 2016, 4 wild polioviruses were detected in Borno state in Nigeria in 3 districts (Jere, Monguno and Gwoza) which were hitherto not accessed, mainly due to insecurity. Nigeria was reclassified as a polio endemic country. High quality outbreak response activities had to be carried out with the implication of innovative technologies to generate evidence that surveillance activities are implemented as planned with high quality data.

The use of tools with mobile devices such as the Auto Visual AFP Detection And Reporting (AVADAR), the Integrated Supportive Supervision (ISS) and the e-Surv (electronic surveillance) contributed to the improvement of the quality of AFP surveillance^6^. AVADAR helps to strengthen AFP surveillance in difficult or hard to reach areas while ISS gives a real-time clear picture of what is going on in the field.

With available new technologies in data management, investigation ofAFP cases is carried out with a mobile device using Open Data Kit (ODK). This data is used to update the initial case investigation form. The updates on the initial investigation are mainly geo localization coordinates that were obtained during on-site case verification, the date of onset of the paralysis and the anatomic site of paralysis. The training of data managers on new software such as ArcGIS, and Tableau has been conducted to strengthen their capacity in producing quality data analysis outputs.

All these innovative strategies aided in estimating the reach of disease surveillance and immunization activities, with evidence, providing geographical coverage which was useful in planning strategies to improve access to these areas not being accessed.

### Feedback and information products

At national, IST and AFRO level, a bulletin or other type of information product is produced after analysis of the data. For the last 2 years, bulletins are systematically produced by AFRO and by IST. The purpose of the bulletin is mainly to monitor surveillance performance indicators and laboratory results including the environmental surveillance results. These feedbacks are shared with countries and partners of the polio eradication programme.

### Capacity building

Since 1999, the Regional office and the ISTs periodically organized a serie of workshops for data managers in the national surveillance units and for national laboratory staff. Participants for each country include the WHO EPI/Surveillance focal point, the data manager in the surveillance unit and the data manager in the laboratory unit where applicable. The main components for those workshops are basic training on EpiInfo and data processing modules for AFP surveillance. At the beginning of the network, this training was carried out annually and a briefing was systematically organized for new staff. Currently the training is done if there are new orientations or modifications of the AFP case investigation form.

## Results

### Data Flow and Sharing

The timeline for sharing data between levels of the system are established in the polio surveillance network. Every week from Monday to Tuesday every country (MOH) shares data with WHO country office. The country office shares with IST from Tuesday to Wednesday, while IST shares with AFRO on Thursday.

Table 1 below summarizes the timeliness of reporting AFP surveillance in West Africa sub region from country to IST level for the period 2014-2017.

Table 1 is an illustration of monitoring timeliness of AFP data reporting in the WHO IST West Africa countries. The same monitoring is carried out in the two other ISTs, Central Africa^1^ (10 countries) and Eastern and Southern Africa^2^ (20 countries).

The acceptable level of timeliness of country reporting AFP data with IST is 80%. In 2014, the overall timeliness of country reporting AFP data with the IST in West Africa was 91%; however, 3 of the 17 countries, namely Guinea Bissau, Mauritania and Sierra Leone, did not achieve the indicator for the year. This indicator remained stable from 2015 to 2017 at approximately 95%. Only Mauritania did not achieve this indicator over the more recent period. In general, it is noted that countries are sensitized on the importance of sharing of AFP surveillance data which is critical given the need for a timely response to each detected polio case^7^.

### Data capture and analysis

**Figure 1:**
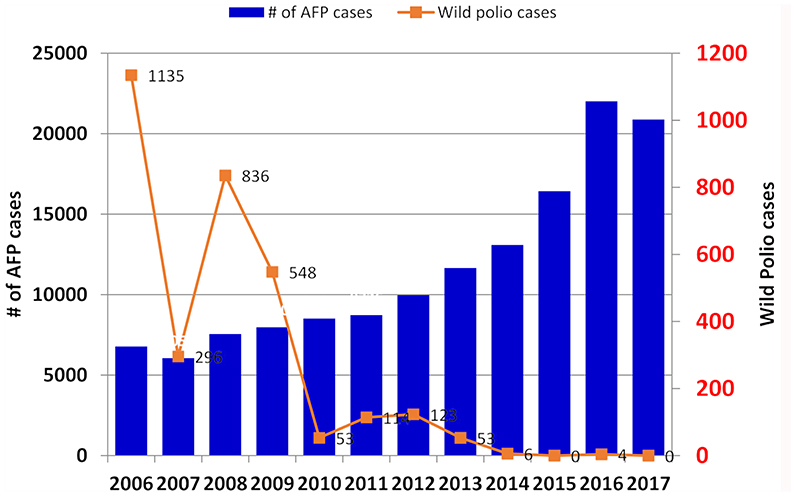
Number of AFP cases and Wild polio cases detected in West Africa by year (2006 – 2017).

Between 2006 and 2017, as shown in Figure 1, there was an increase of the number of AFP cases reported in West Africa while the number of confirmed wild polio virus decreased in the period^8^.

**Figure 2:**
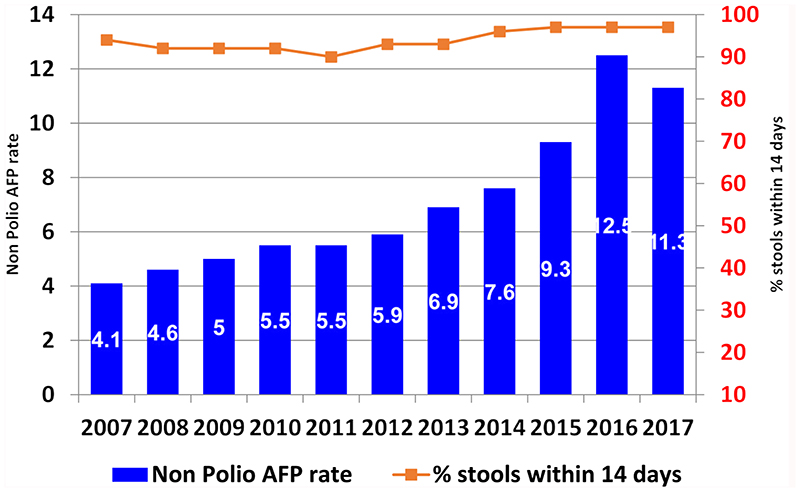
Main surveillance performance indicators by year in West Africa.

Data can be disaggregated by country by province and by district. The analysis on surveillance performance indicators is performed at different levels, country, province and districts. The non-polio AFP rate (target >2/100000 population under 15 years old) and the percentage of stools collected within 14 days (target 80%) are calculated every week in order to detect surveillance performance deficiencies (Figure2). In West Africa for example, the two main surveillance performance indicators have been achieved each year over the 2006 - 2017 period. The non-polio AFP rate remained greater than the target and has increased each year from 2007 to 2016 before decreasing slightly to 11.3 in 2017. The percentage of stools collected within 14 days has consistently been over 90% during the period.

### Data cleaning process

The role of the ISTs and AFRO is to verify data quality by checking that variables in the database are completed, that inconsistencies are corrected as well as monitoring the timeliness and completeness of AFP data reporting. Feedback on data quality is routinely provided to countries. To minimize inconsistencies and incompleteness in the database, AFRO/IST encourages the organization of monthly data harmonization meetings. The meetings focus on vaccine preventable diseases data and bring together staff from the EPI program, surveillance, laboratory and partners. Data is reviewed to ensure that discrepancies are eliminated, and errors corrected.

Table 2 below is an illustration of the number of data quality issues per variable and geographical zone addressed during data harmonization meeting in Nigeria. The number of doses of OPV received by the child, the dates of stool collection and the date of onset of paralysis were the variable with the high number of quality issues.

The aim of the exercise is the completeness, consistency and integrity of data. The errors or empty variables detected by the national level are corrected as participants have necessary documents to provide the information to update the database. From month to month the quality of data improves and the number of incomplete or incorrectly filled data items is reduced. As a result, at the end of the year, the polio dataset contains a minimum of missing or inconsistent variables.

### Integration of data from different sources and use of innovative technologies

AFP surveillance data, polio laboratory data and vaccination coverage can be integrated to provide a more comprehensive epidemiological analysis. This analysis is further enhanced through the use of innovative technologies such as the Integrated Supportive Supervision platform which indicates where surveillance activities are carried out.

**Map 1:**
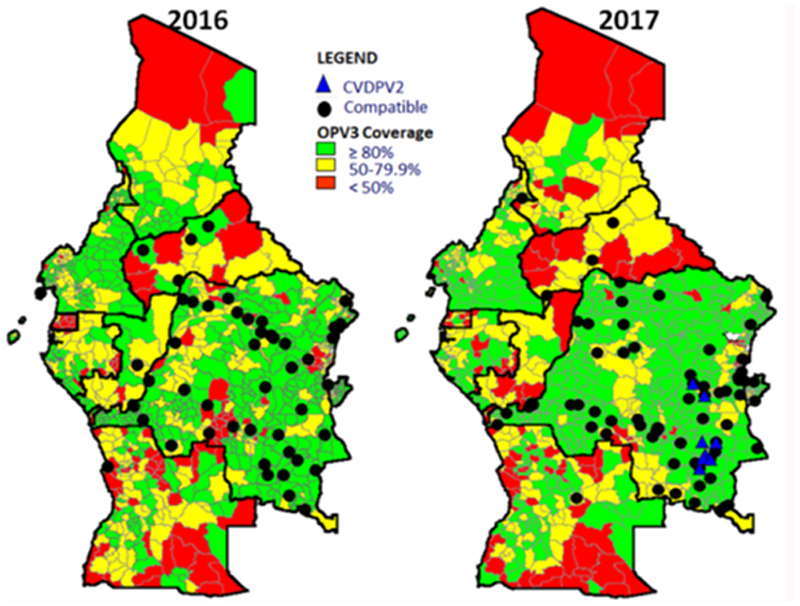
AFP Detection and OPV3 coverage in 2016 and 2017, Central Africa

Map1 shows the AFP surveillance performance indicators in 2016 and 2017 with the third dose of Oral Polio Vaccine (OPV3) vaccination coverage for countries in IST Central Africa. These maps show that vaccination data can be questioned. In the case of DRC in 2017, cVDPV2 cases appear to be notified in areas that recorded good vaccination coverage. For each case with a final classification as compatible with polio, geo coordinates have been added in the case investigation form via the ISS platform. It is therefore possible to have the exact location of the AFP case. In 2017, cases of cVDPV2 were identified and shown on the map.

At Global level, there is Polio Information System (POLIS), a centralized database integrating data from all WHO regions. Polio data from African Region is integrated every week. This system provides standard set outputs^9^.

### Feedback and information products

**Figure 3:**
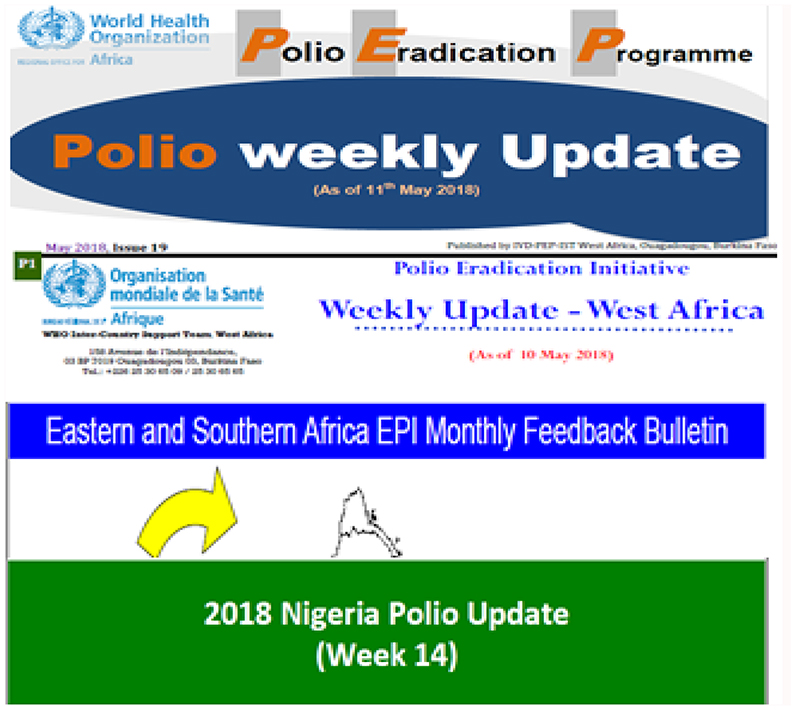
Feedback and information products in AFRO, in IST and in country

Feedback is necessary to provide information on surveillance performance indicators to the field and aids in monitoring and implementation of corrective actions. The focus is on data quality, surveillance performance indicators and the timeliness and completeness of reporting. The feedback is published weekly by the ISTs, by AFRO and individually by the large countries such as Nigeria. Integrated feedback bulletins combining polio with other vaccine preventable diseases’ information are also published at country level on monthly basis. Figure 3 shows samples produced by AFRO, IST and Nigeria.

## Discussions

### Data quality

The data collection system constructed to support the polio program objectives has provided critical information to progress towards eradication. Since introduction of the use of mobile device and scale up of digital innovations in early 2014, continuous capacity building was a key component that led to data quality improvement. Surveillance performance and timeliness of reporting have improved in almost all the countries. There have been remarkable improvements in timeliness of case detection, reporting and investigation, with the use of mobile devices. This has directly impacted the key surveillance indicators as shown by the analysis of various performance indicators.

Data harmonization meetings in countries are an effective forum to reconcile lab, surveillance and immunization data. As a result, data is used at all levels for better planning and guiding appropriate interventions to correct areas with poor surveillance performance. The success of the system can be attributed to national EPI focal points and Data managers who are trained and possess computer and software to allow sharing complete data in a timely manner. However, there are still multiple data entries from field to surveillance unit and the laboratory.

### Data management tools

With the standardization of the variables to be collected, it is easy to merge data coming from countries into regional level database. The data collection form and the data processing tools are the same in all countries of Africa Region. The forms and SOPs are occasionally modified at AFRO level after a global consensus. Final documents are shared with countries with the modified data entry interface. The timeline for sharing data from countries to WHO Regional Office are well defined. Feedback bulletins are produced at all levels.

### Surveillance

AFP surveillance performance indicators are well defined and monitored. Surveillance data entry and transmission is done every week. The monitoring of system performance is done by reviewing indicators as updated weekly. The system has allowed the notification of an increasing number of AFP cases. However, to sustain this level of indicators, there is a continuous need to maintain trained personnel and financial resources to carry out activities in the field.

### Introduction of new technologies

New information technologies including the use mobile devices for data collection and GIS have been incorporated into the polio surveillance system^10^. Since 2016, all AFP case investigations require that the location of the investigation is captured. Other issues associated with paper-based data collection such as multiple data entries and possible errors at the data management level have also been addressed with the use of mobile devices, affording more time to concentrate on data analysis. Continuous data analysis permits the identification of non-reporting, silent areas and areas having weak surveillance performance so corrective actions can be taken. In general, new technologies are used in selected districts in countries due to the high cost of implementation.

## Limitations

Some of the components are described in the surveillance manuals and user guides for the system. The funding part of activities carried out is not addressed in this paper. This can be published in another article. This article is focused only on surveillance activities carried out after the World Health Assembly in 1988. The strategies were not initiated or deployed at the same time. Implementation of innovative solutions since 2014, began with the usage of Magpi and ODK for monitoring and generating evidence of surveillance impact. Since 2017, community-based surveillance initiatives such as AVADAR and eSurveillance have been introduced in a phased manner throughout the AFRO region to improve the sensitivity of AFP surveillance as well as active case-search for vaccine-preventable diseases.

The review of process indicators and supporting on-the-job training has shown to considerably improve surveillance indicators. AVADAR has been deployed in 11 countries (Nigeria, Sierra Leone, Liberia, Chad, Cameroon, Niger, DRC, South Sudan, Burkina Faso, Mali and CAR) in the AFRO region between 2016 and 2019 while eSurveillance has been deployed across the region in phases. Although implemented to a limited extent and in selected countries, these initiatives have contributed to improving the overall data management system in the region.

## Conclusions

Polio eradication Programme is at a crucial stage to achieve its objectives by 2023. Data quality is critical for decision making and action. Sustained efforts over a long period have been made towards improving the quality of data in the Africa Region. These efforts have resulted in improved timeliness of data reporting, provision of monitoring tools, capacity building of staff involved in surveillance, data management and laboratory and the use of new technological innovations. A strong information system has been put in place from the community level to the global level with a link between surveillance, laboratory and immunization coverage data. Feedback provided at all levels is used to improve programme performance. Innovations including geolocalization of cases and the use of smartphones for data reporting have also improved system performance. To maintain standards in Polio Information system, there is need for continuous training of the staff on areas of surveillance, information systems, data analysis and information sharing. The use of innovative technologies on web-based system and mobile devices with validation rules and information check will avoid multiple entries.

The scaling up of the innovations with consistent monitoring of the analysis of surveillance performance indicators will be critical as the Region approaches the objective of polio eradication certification.

1. IST Central Africa countries are Angola, Burundi, Cameroon, Central African Republic, Chad, Congo, Democratic Republic of Congo, Equatorial Guinea, Gabon and Sao-Tome & Principe.
2. IST Eastern and Southern countries are Botswana, Comoros, eSwatini, Ethiopia, Eritrea, Kenya, Lesotho, Madagascar, Malawi, Mauritius, Mozambique, Namibia, Rwanda, Seychelles, South Africa, South Sudan, Tanzania, Uganda, Zambia and Zimbabwe.

## Figures and Tables

**Table 1:** Timeliness of AFP data reporting by country in West Africa, 2014-2017

	2014	2015	2016	2017
ALGERIA	96%	94%	94%	84%
BENIN	100%	100%	100%	100%
BURKINA FASO	98%	98%	100%	100%
CABO VERDE	81%	96%	91%	92%
COTE D’IVOIRE	100%	100%	100%	100%
GAMBIA	98%	98%	98%	92%
GHANA	90%	92%	81%	80%
GUINEA	92%	89%	98%	96%
GUINEA BISSAU	73%	87%	87%	96%
LIBERIA	100%	100%	100%	100%
MALI	96%	98%	100%	100%
MAURITANIA	62%	74%	66%	75%
NIGER	100%	96%	100%	96%
NIGERIA	100%	100%	100%	100%
SENEGAL	96%	100%	100%	100%
SIERRA LEONE	69%	92%	92%	88%
TOGO	98%	100%	100%	96%
West Africa	91%	95%	95%	94%

**Table 2:** Errors corrected during data harmonization meetings on selected variables in Nigeria

Variable	NORTH CENTRAL ZONEs	NORTH EAST ZONE	NORTH WEST ZONE	SOUTH EAST ZONE	SOUTH SOUTH ZONE	SOUTH WEST ZONE	NATIONAL
Date of Birth	0	0	0	0	0	0	0
Sex	0	2	0	0	0	0	2
True AFP Status	1	1	1	0	0	0	3
Date Case Investigated	0	2	5	0	0	0	7
Date 2^nd^ stool collected	0	1	10	0	0	0	11
Total Polio doses	6	10	0	0	0	0	16
Date of Onset	0	2	8	0	0	0	10
Date 1^st^ stool collected	0	1	10	0	0	0	11

## List of Abbreviations

AFP: Acute Flaccid Paralysis
AVADAR: Auto Visual AFP Detection and Reporting
CAR: Central African Republic
CV: Vaccination Coverage (quoted in French)
cVDPV: Circulating Vaccine Derived Polio Virus
DRC: Democratic Republic of Congo
EPI: Expanded Program on Immunization
e-Surv: electronic surveillance
GIS: Geographic Information System
GPEI: Global Polio Eradication Initiative
ISS: Integrated Supportive Supervision
IST: WHO Inter-Country Support Team Office
IVD: Immunization and Vaccine Development
MOH: Ministry of Health
ODK: Open Data Kit
OPV: Oral Polio Vaccine
OPV3: Third dose of OPV (given at 14 weeks of age)
PEI: Polio Eradication Initiative
PEP: Polio Eradication Programme
POLIS: Polio Information System
SOPs: Standard Operating Procedures
WCO: WHO Country Office
WHA: World Health Assembly
WHO/AFRO: WHO Regional Office for Africa
